# Dietary diversity practice and its influencing factors among pregnant women in Afar region of Ethiopia: mixed method study

**DOI:** 10.1186/s12884-022-04641-y

**Published:** 2022-04-06

**Authors:** Temesgen Gebeyehu Wondmeneh

**Affiliations:** Department of Public Health, College of Health Science, Samara University, Semera, Ethiopia

**Keywords:** Dietary, Diversity, Factor, Pregnant, Women

## Abstract

**Background:**

Pregnancy can aggravate nutritional deficiencies, especially micronutrient deficiencies, which can have major health impact for the fetus and mother. Women in low-income countries are frequently malnourished when they become pregnant. Identifying the magnitude of dietary diversity and its influencing factors among pregnant women in the pastoral region of Afar, where no study has been conducted, is critical for establishing an intervention program in the region.

**Method:**

A mixed study comprising 241 pregnant women and six focus group discussions was conducted from October 1 to November 10, 2018. Participants in the quantitative study were selected by a systematic sampling method, whereas those in the focus group discussions were selected by a purposive sampling method. The data were collected using pretested questionnaires administered via face-to-face interviews. Logistic regression determines the association between the dietary diversity practice and its influencing factors. The results were presented by the odds ratio with a 95% confidence interval. A *P*-value of < 0.05 is used to declare a statistically significant. A thematic framework was used to analyze the qualitative data.

**Results:**

Seventy-three percent of pregnant women had poor dietary diversity. Dietary diversity was higher in younger pregnant women who were under the age of 20 years (AOR = 5.8; at 95% CI: 1.6–13.5) and aged between 21 and 25 years (AOR = 3.9; at 95% CI: 1.1–12.2) than those pregnant women over the age of 30 years. Those participants with a high average family income (above 4500 birr) had good dietary diversity compared to those with an average family income of less than 1500 birr (AOR = 0.1: 95% CI; 0.02–0.7) and 1500–3000 birr (AOR = 0.05: 95% CI; 0.01–0.2). Pregnant women who had one antenatal care visit had less dietary diversity practice than those who had four or more antenatal care visits (AOR = 0.18: 95% CI; 0.04–0.8). Protein-rich foods (meat and eggs), cereal-based semi-solid foods (porridge and soup), milk, bananas and cabbage, were the most commonly tabooed foods during pregnancy. Protein-rich foods were thought to increase the size of the fetus. Semi-solid foods (porridge and soup), bananas, and cabbage, on the other hand, were thought to stick to the fetus's body.

**Conclusion:**

Most of the study participants had poor dietary diversity. Older women have lower dietary diversity practices than younger women. Pregnant women with a low family income and only one prenatal care visit were less likely to practice dietary diversity than pregnant women with a high family income and those with four or more antenatal care visits. Pregnant women practiced food taboos due to misconceptions that were producing large babies and attached to the babies’ bodies. A public health campaign emphasizing the necessity of antenatal care follow-ups should be launched. Community nutrition education should be provided to reduce the traditional beliefs about certain foods, especially for older women.

## Background

Dietary diversity refers to the number of food groups consumed over a period of time [1] and is a nutrient adequacy indicator [2, 3]. Nutrient deficiency or excess disrupts metabolism. Intake of low-energy–density but high-nutrient diversity diets can be the key to promoting and maintaining optimal health [4]. Major pregnancy risks such as maternal and neonatal mortality, intrauterine growth retardation, preterm delivery, low birth weight, and infectious risk can be reduced by proper nutrition [5]. Caribbean, Central, and South American women consumed more calories, fat, protein, and carbohydrates than African and Asian women. Across these regions, cereal-based foods constituted the majority of people's diets [6]. Exogenous household shocks and intra-household food allocation decisions have a negative impact on women's diets. Women's diets in rural India are less diverse than those of the rest of the family [7]. Thirty-seven percent of pregnant women in South Africa and a comparable 37% of pregnant women in Eastern Nigeria practiced dietary taboos, such as not eating meat and egg, are due to cultural beliefs linked with the presumption of difficulty in delivering [8, 9]. In a study in Kenya, the mean dietary diversity score was 6.84 ± 1.46, with a minimum dietary diversity of 98%. Cereal foods were the most popular food group (99%) [10]. In some district level studies of Ethiopia, the magnitude of poor dietary diversity was 55.2% in the Bale Zone [11] and 54.8% in Dessie [12]. In a study of Northeast Ethiopia, the mean dietary diversity score was 4.45 ± 1.32. Fifty-seven percent of pregnant women had poor dietary diversity. All of the study participants ate starch-based staple foods. Other vegetables were consumed by almost all the respondents (98.7%). About 66.3% of women ate plant-based foods from nuts and pulses, 55.3% consumed other vitamin A-rich fruits and vegetables, and 46.6% took milk and milk products [13]. Dietary diversity was influenced by educational levels, monthly family income, antenatal care (ANC) visits, and nutritional counselling [14, 15]. Furthermore, dietary diversity was determined by occupation, food insecurity, and eating less frequently [16]. Green leafy vegetables, milk products, sugarcane, green peppers [17], meat, bread, and cold water [18], were taboos for pregnant women. The reason for banning these foods was fear of the big fetus, which is linked to the difficulty of delivery.

Although few studies have been conducted in other parts of Ethiopia, the practice of dietary diversity and its influencing factors among pregnant women have not been studied in the context of the Afar pastoral region, where socio-cultural and economic realities are considerably different. This study determined the magnitude of dietary diversity practice and its influencing factors among pregnant women residing in Awash Seven District, Afar Region of Ethiopia.

## Methods

### Study area

The study was conducted at the Awash seven health facility in the Afar region of Ethiopia. Large proportions of the Afar people are pastoral and follow the Muslim religion. The region's climate is dry and hot, and food shortages occur annually. Aid organizations assisted almost all vulnerable people under the Productive Safety Net Programme (PSNP) in Ethiopia, which began in 2005 [19]. Goats, sheep, and camels are the most common livestock used by households. The Afar social structure is based on descent and affine relationships. The Afar have patrilineal descent system that assigns a person to a certain clan (the society called mela) [20].

### Study design and sample size determination

A mixed study design was used to determine dietary diversity practice and its influencing factors among pregnant women in the Afar region of Ethiopia. The duration of quantitative data collection was from October 1 to November 5, 2018, while the qualitative data collection period was from October 6 to October 10, 2018. The sample size for quantitative data was calculated using a single proportion formula. Thus, considering 95% confidence level with 5% precision, and taking 57% inadequate dietary diversity from a prior study in Dire Dawa, Eastern Ethiopia [13], and the sample size was 376. However, in the Afar region, pregnant women who received antenatal care follow-ups were rare, so selecting 376 pregnant women from 512 took effort and money. As a result, the correction formula [21] was again used to calculate the representative sample. Based on this, the sample size was 217, and after adding 15% non-response rate, the final sample size was 250. Focus group discussions (FGDs) with pregnant women were conducted until the qualitative data was saturated.

### Inclusion and exclusion criteria

All pregnant women who agreed to take part in the study and had lived in the study area for at least one year before the study period were included. The study excluded pregnant women who had chronic diseases like cancer or diabetes because these illnesses are known to impact a person's food consumption and nutritional status. The respondents' health information was used to compile this data. Pregnant women who had consumed special diets in the previous 24 h owing to holidays or celebrations were also excluded.

### Sampling technique and procedures

The sampling interval was determined by dividing the total number of pregnant women who attended antenatal care in the health facility three months ago by the total sample size. The sampling interval (k) was approximately two. Then, using a systematic sampling technique, the required study subjects were selected. From the first two study subjects, the first study subject was selected by lottery, and then every second study subject was selected until the required sample size was achieved. However, pregnant women who had previously participated in the study were not re-interviewed. Purposive sampling was used to select focus group discussants (FGDs) for the qualitative study. The FGD participants, on the other hand, were not the same as those who were sampled for quantitative data.

### Data collection instruments and procedures

For quantitative data, data on demographic, socio-cultural, and economic factors as well as maternal health service utilization factors were constructed from previous literature and collected by face-to-face interview using pre-tested structured questionnaires. Three data collectors (two midwives and one nurse) were trained to collect quantitative data. The training allowed them to become familiar with the food groups and particular foods within each food group, allowing them to place recalled foods into the appropriate food groups. Data on dietary diversity was collected using a modified individual dietary diversity score (IDDS) questionnaire developed by the food and agriculture organization (FAO) [22]. The individual dietary diversity score (IDDS) questionnaire consisted of nine food groups. All the data collectors were fluent in Afar (native). The questionnaires are first written in English and then translated into the Afar language by a third person who is a native Afar speaker with translation experience. Health extension workers in the community and midwives at the Awash seven health center antenatal care (ANC) clinic were also consulted to amend the food lists in the food groups, whether they matched with local names of foods and acceptable terminology was agreed upon (modification was carried out based on local language). The respondents were asked to recall all foods (meals and snacks) consumed the previous day and night (a 24-h recall). After recalling all foods and beverages consumed, these food items were recorded. The interviewers underlined the corresponding foods in the list under the appropriate food categories and entered "1" in the column next to the food group if at least one food was consumed. Once the recall is completed, look for food groups that were not consumed by the respondents. After ensuring that no meals from that food group were eaten, "0" was filled in the right-hand column corresponding to the food group.

Two data collectors (two nurses) were recruited and trained to collect qualitative data. The data was collected by focus group discussion using open-ended guiding questions that were developed based on the study objective. The purpose of the study was explained to each study participant before data collection started for both quantitative and qualitative data. Those who agreed to take part in the study were then interviewed face-to-face in a quiet, comfortable, and convenient location in the health facility in order to better understand each other and ensure confidentiality. The handwritten field notes from all of the focus groups were transcribed into Microsoft Word 7, and everything was translated from Afar to English, including certain key Afar words in brackets. The supervisor checked the collected data for completeness and accuracy daily. The guiding questions used for interview focus group discussions are shown in Table 1.

**Table 1:** Show the key questions used in focus group discussion interviewing guidelines

Participants	Key questions
Pregnant women	Are there certain foods that a pregnant woman should avoid eating in order to protect herself and her unborn child? What are these dietary taboos, exactly?

### Variables and operational definition

Independent variables were age, religion, ethnicity, marital status, residence, family size, educational status, occupation, average monthly income, birth interval, parity, month of pregnancy, antenatal care visits, food taboos, and frequency of eating per day. The dependent variable was dietary diversity (poor or good).

Poor dietary diversity was considered when pregnant women consumed the lowest dietary diversity (≤ 3 food groups) [23], whereas pregnant women who consumed more than three food groups were considered to have good dietary diversity.

### Data analysis

SPSS Version 23 was used for the analysis. Tables with frequencies were used to present the results. Binary logistic regression was used to find significant associations between dietary diversity practice and independent variables. Variables having a *p*-value less than 0.05 in binary logistic regression were included in multivariate logistic regression. The regression analysis's results were provided as odd ratios (OR), with a 95% CI and a significance level of less than 0.05. When respondents consumed four or more food groups, their responses were categorized as "good," and if they consumed fewer than four food groups, their responses were categorized as "poor." The qualitative data was manually analyzed using the framework techniques in multidisciplinary research [24]. The results of the thematic analysis are presented in the form of a narrative with supporting quotes. Finally, the qualitative study's findings were triangulated with the quantitative findings.

### Ethical consideration

The Ethics Committee at Samara University's Health Science College gave ethical clearance. Before recruiting, informed consent was obtained from each mother after explaining the study's objectives. Participation in the study was entirely voluntary. All information was kept in strict confidence.

## Results

### Quantitative study results

#### Socio-demographic characteristics of pregnant women residing in Awash seven district, Afar region of Ethiopia

The response rate of the study was 96.4% (241/250). About 29.9% of participants were in the age range of 21–25 years. Ninety-five percent of participants were married. Most pregnant women (72.2%) follow the Islamic religion. More than half of the study participants were Afar ethnicity (58.5%). Almost three-fourths (75.9%) of the participants were urban residents. Illiterate pregnant women represented 35.3% of the study participants. Housewives accounted for nearly three-quarters (75.5%) of pregnant mothers. Twenty-seven percent of pregnant women lived with extended family members (> 4 family members). Nearly half (50.6%) of study participants had an average family income of between 1500 and 3000 birr (Table 2).

**Table 2:** Shows socio-demographic characteristics of pregnant women in Awash Seven district, Ethiopia

Variables	Categories	No (%)
Age	≤ 20	51 (21.2)
	21–25	72 (29.9)
	26–30	66 (27.4)
	≥ 31	52 (21.6)
Marital status	Married	229 (95)
	Unmarried	12 (5)
Religious	Orthodox	55 (22.8)
	Protestant and catholic	12 (5)
	Muslim	174 (72.2)
Ethnicity	Others	39 (16.2)
	Amhara	61 (25.3)
	Afar	141 (58.5)
Residence	Urban	183 (75.9)
	Rural	58 (24.1)
Educational status	Illiterate	85 (35.3)
	Primary school	68 (28.2)
	Secondary school	63 (26.1)
	College and above	25 (10.4)
Occupation	Housewives	182 (75.5)
	Government employed	23 (9.5)
	Merchants	36 (14.9)
Family size / members	Four and below	176 (73)
	Above four	65 (27)
Average monthly income (Ethiopian birr)	< 1500	53 (22)
	1500–3000	122 (50.6)
	3001–4500	33 (13.7)
	> 4500	33 (13.7)

Others represent Oromo, southern nation and nationalities peoples, and Tigray peoples

#### Obstetric and health service utilization characters of pregnant women residing in the Awash seven district, Afar region of Ethiopia

About 67.3% of multiparous pregnant women had a birth interval of more than two years. Nulliparous participants accounted for 32.4% of the total study participants. Almost half of the study participants (49.8%) were longer than six months pregnant. Only 7.1% of study participants had four antenatal care visits, while 49% of pregnant women had one antenatal care visit (Table 3).

**Table 3:** Shows obstetric and health service utilization characters of pregnant women in Awash Seven district, Ethiopia

Variables	Categories	No (%)
Birth interval	≤ 2 years	53 (32.7)
	> 2 years	109 (67.3)
Parity	Nulliparous	78 (32.4)
	Multiparous	163 (67.6)
Month of pregnancy	Less than 3 months	38 (15.8)
	3–6 months	83 (34.4)
	Greater than 6 months	120 (49.8)
Antenatal care visits	One	118 (49)
	2–3 times	106 (44)
	≥ 4 times	17 (7.1)

#### Dietary habit of pregnant women residing in Awash seven district, Afar region of Ethiopia

Seventy-three percent of study subjects had poor dietary diversity practice, whereas the remaining participants had good dietary diversity practice. Only 9.5% of pregnant women consumed meals five times a day, whereas 74.2% of study participants ate three times a day. Around thirty-seven percent (37.3%) of pregnant women had food taboos during their pregnancy. During pregnancy, 58.9% of women avoided at least one of the protein foods (meat and eggs), while 13.3% avoided at least one of the semi-solid foods (cereal soup and porridge) (Table 4).

**Table 4:** Shows dietary habit of pregnant women in Awash Seven District, Ethiopia

Variables	Categories	No (%)
Dietary diversity	Poor	176 (73)
	Good	65 (27)
Frequency of food taking per day	Three times	178 (74.2)
	Four times	40 (16.7)
	Five times	22 (9.2)
Are there certain types of food taboos?	Yes	90 (37.3)
	No	151 (62.7)
Types of foods avoided during pregnancy	Reason for prohibition	
Protein (meat and egg)	Producing a big fetus	53 (58.9)
Semi-solid foods (porridge and cereal made soup)	Attached to the fetus body	12 (13.3)
Milk	Producing a large fetus and attaching to the fetus's body	11 (12.2)
Fruit and vegetable (banana and cabbage)	Attached to the fetus body	7 (7.8)
Carbohydrate (bread and other sweet foods)	Producing big fetus	7 (7.8)

#### Pregnant women consumed food groups in the previous 24 h in Awash seven district, Afar region of Ethiopia

The overall mean of the individual dietary diversity scores was 3.1 ± 1.38. The mean value of the poor and good dietary diversity scores was 2.44 ± 0.6 and 5 ± 1.075, respectively. Over ninety percent of participants (91.3%) ate starchy staple foods **(***eragrostis tef*, wheat, and corn). About 61.8% of pregnant women consumed legumes, nuts, and seeds. More than half of the participants (57.7%) took milk and milk production (Table 5).

**Table 5:** Shows the types of food groups consumed by pregnant women in the previous 24 h in Awash seven district, of Ethiopia

Dietary diversity score	Mean ± S.D
The overall mean of dietary diversity score	3.1 ± 1.38
The mean of poor dietary diversity score	2.44 ± 0.6
The mean of good dietary diversity score	5 ± 1.075
Food groups	No (%)
Starchy Staple (cereal based like eragrostis tef, wheat and corn)	220 (91.3)
Dark green leafy vegetables (kale and spinach)	44 (18.3)
other vitamin A rich foods and vegetables (mango, papaya and orange)	61 (25.3)
Other fruits and vegetables (avocado, lemon, cabbage, banana and apple	61 (25.3)
Organ meat	5 (2.1)
Meat and fish	35 (14.5)
Eggs	41 (17)
Legume, nuts and seeds	149 (61.8)
Milk and milk products	139 (57.7)

*SD* standard deviation

#### Factors influencing pregnant women's dietary diversity practices in Awash seven district, Afar region of Ethiopia

When confounding factors were adjusted, participants under the age of 20 years (AOR = 5.8; at 95% CI: 1.6–13.5) and those aged 21–25 years (AOR = 3.9; at 95% CI: 1.1–12.2) had better dietary diversity practices than those over the age of 30 years. Participants with less than 1500 birr in monthly family income (AOR = 0.1; 95% CI: 0.02–0.7) and those with 1500–3000 birr in monthly family income (AOR = 0.05; 95% CI: 0.01–0.2) consumed less dietary diversity than those with more than 4500 birr. Pregnant women who had one antenatal care visit were 82% more likely to have poor dietary diversity consumption than those who had four or more antenatal care visits (AOR = 0.18; 95% CI: 0.04–0.8) (Table 6).

**Table 6:** Shows influencing factors associated with dietary diversity practices among pregnant women in Awash seven district, Ethiopia

Variables		Dietary diversity		COR with 95% CI	AOR with 95% CI
		Poor	Good		
		No (%)	No (%)		
Age (in years)	≤ 20	31 (12.9)	20 (8.3)	**3.1 (1.2–7.7)**	**5.8(1.6–13.5)**
	21–25	49 (20.3)	23 (9.5)	2.2 (0.9–5.4)	**3.9 (1.1–12.2)**
	26–30	53 (22)	13 (5.4)	1.2 (0.5–3.0)	1.6 (0.4–6.1)
	≥ 31	43 (17.8)	9 (3.7)	1	1
Religious	Orthodox	32 (13.3)	23 (9.5)	**2.5 (1.3–4.7)**	
	Protestant & catholic	9 (3.7)	3 (1.2)	1.2 (0.3–4.5)	
	Muslim	135 (56.2)	39 (16)	1	
Residence	Urban	125 (52)	58 (24)	**3.4 (1.5–7.9)**	
	Rural	51 (21.2)	7 ( 2.9)	1	
Educational status	Illiterate	75 (31.1)	10 (4.1)	**0.1 (0.03–0.3)**	
	Primary school	51 (21.2)	17 (7.1)	**0.2 (0.1–0.6)**	
	Secondary school	40 (16.6)	23 (9.5)	0.4 (0.1–1.0)	
	College and above	10 (4.1)	15 (6.2)	1	
Occupation	Housewives	143 (59.5)	39 (16)	**0.4 (0.2–0.9)**	
	Government employed	11 (4.6)	12 (5)	1.7 (0.6–4.9)	
	Merchants	22 (9.1)	14 (5.4)	1	
Monthly family income (ETB)	< 1500	43 (17.8)	10 (4.1)	**0.1 (0.03–0.3)**	**0.1 (0.02–0.7)**
	1500–3000	107 (44.4)	15 (6.2)	**0.1 (0.02–0.1)**	**0.05 (0.01–0.2)**
	3001–4500	17 (7.1)	16 (6.6)	**0.3 (0.1–0.9)**	0.6 (0.1–2.4)
	> 4500	9 (3.7)	24 (10)	1	1
Antenatal care visits	One	99 (41.1)	19 (7.9)	**0.17(0.1–0.5)**	**0.18 (0.04–0.8)**
	2–3 times	69 (29)	37 (15)	0.5 (0.2–1.3)	0.9 (0.2–3.9)
	≥ 4 times	8 (3.3)	9 (3.7)	1	1
Frequency of eating per day	Three times	147(61)	31 (13)	**0.12 (0.1–0.3)**	
	Four times	21 (8.8)	19 (7.9)	0.5 (0.2–1.5)	
	Five times	8 (3.3)	14 (5.8)	1	
Food prohibition	Yes	79 (32.8)	11(4.6)	**0.25 (0.1–0.5)**	
	No	97 (40.2)	54(22.4)	1	

### Qualitative study results

In the qualitative study, 38 pregnant women were involved in six focus group discussions, four of which were held with urban residents and two with rural residents.

In urban residents, seven pregnant women participated in each of the two focus group discussions, and six study participants involved in the remaining two focus group discussions. For rural residents, each of the two focus group discussions had six participants. Most participants (44.7%) were between the ages of 21–30 years, with the remaining 15.8% and 39.5% being under and over the age of 20 years and 30 years, respectively. About 28.9% and 44.7% of participants were illiterate and in primary school, respectively, while 18.4% and 7.9% of pregnant women were in secondary school, college and above, respectively. Ninety-nine percent (89.5%) of pregnant women were multiparous. More than half of the participants (55.3%) were pregnant for 3–6 months, and 34.2% were pregnant for longer than six months. Half of the study participants had one prenatal care follow-up, while 42.1% had two or three antenatal care follow-ups. In this qualitative study, dietary taboos during pregnancy were investigated among pregnant women. According to focus group discussants, the most commonly avoided foods during pregnancy were semi-solid and solid cereal-based foods, fruit and vegetables, animal products, and soft drinks. A key theme, food taboos, was developed using codes and categories (Table 7).

**Table 7:** Shows the development of a key theme, food taboos during pregnancy, using codes and categories based on reports from pregnant women reside in Awash district, Afar region of Ethiopia

A major theme	Categories	Codes
Food taboos during pregnancy	Semi-solid and solid foods	Porridge, soups, bread
	Fruits and vegetable	Banana, cabbage
	Animal products	Milk, meat, eggs
	Soft drink	Coca cola, sprit

The following are the details of the qualitative study's participant interviews

During pregnancy, most participants believed that semi-solid foods, as well as some fruits and vegetables, should be avoided because they thought these foods would stick to the fetus's body. A 35-years-old woman explained that "porridge, cereal soup (locally known as atimt), bananas, and cabbages were not consumed during pregnancy since they could cling to the fetus' body" (a 35-years pregnant woman in FGD1).

Most group members reflected their opinion that sweat foods and animal products should not be consumed by pregnant women since the fetus would grow large and be difficult to deliver. A 28-year-old pregnant woman stated, "In our society, pregnant women do not consume meat, eggs, and milk since these foods produce large fetuses and cause delivery to be delayed" *(a 28-year pregnant woman in FGD2).*

Participants in the focus group discussion (FGD) also avoided milk, fruits, and semi-solid foods when pregnant. A pregnant 29-year-old woman expressed her belief that "yogurt, bananas, and porridge were adhering to the unborn fetus' body, resulting in an abnormal child. Thus, I did not eat these foods "(a 29-year-old pregnant woman in FGD3).

Almost all the participants in the focus group discussion said that protein and carbohydrate diets were forbidden during pregnancy. This was explained by a 37-year-old woman who stated that "I did not consume meat, eggs, or bread because they increased the weight of the fetus and caused prolonged labor and bleeding" (a 37-year-old pregnant woman in FGD4).

To prevent fetal obesity and facilitate delivery, coffee with milk and sugar was not allowed to be consumed by pregnant women. A 39-year-old pregnant woman clarified this: "in Afar culture, a mixture of coffee, milk, and sugar (locally known as ashara) is not taken during pregnancy since it increases fetal weight and makes it difficult to easily deliver" (a 39-year-old pregnant woman in FGD5).

The majority of group respondents avoided soft drinks and semi-solid foods during their pregnancy due to concerns about a thin baby bone caused by soft drinks and the attachment of semi-solid foods to the fetus's body. A 30-year-old woman noted that "Coca-Cola and Sprite made the fetus bone thin, and porridge and cereal soup (locally called atmit) stuck to the fetus body. So, we did not consume these food items "(a 30-year-old pregnant woman in FGD6).

## Discussion

Inadequate dietary diversity can harm both the mother and the fetus, with the effects on the fetus perhaps persisting into childhood. Recognizing pregnant women's dietary diversity practices is critical for promoting maternal nutrition, health, and child development [4, 5]. Dietary diversity is affected by a variety of internal and external risk factors [7]. There is no study on the dietary diversity among pregnant women in the Afar region of Ethiopia. Thus, identifying the magnitude of dietary diversity practice and its influencing factors among pregnant women in the pastoral region of Afar is merits for policy and program consideration. The average dietary diversity score in this study was 3.1 ± 1.38, which is lower than studies in Kenya [10] and Dire Dawa, northeast Ethiopia [13]. Seventy-three percent of study participants exhibited poor dietary diversity. These findings were higher than those of a study in Kenya [10] and in different district-level studies in Ethiopia [11–13]. The discrepancy in dietary diversity scores could be attributable to the study area, where more cultural practice might influence food intake during pregnancy, as opposed to studying settings in major urban areas, where urban inhabitants could have more nutrient diversity. In the current survey, 37.3% of pregnant women avoided certain types of foods. This finding is consistent with the findings in South Africa [8] and Eastern Nigeria [9]. Dietary diversity is an indicator of nutrient adequacy [2, 3]. Therefore, the lack of adequate dietary diversity in most pregnant women in the current study suggested nutrient deficiency, implying that the requirement for sufficient energy and key nutrients was not met. Most participants (91.3%) ate cereal-based diets. This result is consistent with study in low and middle-income countries [6], but lower than a study in Kenya [10] that found cereal-based foods were the most popular (99%). Moreover, all food groups consumed by pregnant women in the current study were lower than in a study done in Northeast Ethiopia [13] except milk and milk products, which were common in the pastoral region of Afar. Meat and eggs were the most avoided foods during pregnancy and the least consumed food groups out of the nine food groups. These findings strengthen the qualitative finding, which revealed that animal products (meat and eggs) were the most popular foods avoided during pregnancy. This evidence conforms to studies in South Africa [8], Eastern Nigeria [9], and Ethiopia [18]. Moreover, according to study participants in both quantitative and qualitative studies, semi-solid foods (porridge and cereal soup), some fruits and vegetables, milk, and carbohydrate foods (bread and sweet foods) were also forbidden during pregnancy. These findings are consistent with a prior study conducted in Ethiopia [17]. Fear of fetal weight increase, which is linked to delivery difficulty and the assumption that semi-solid meals, fruit, and vegetable attachments to the fetus' bodies were the reasons for avoiding eating these foods. Participants under the age of 20 years and those between the ages of 21 and 25 years consumed better dietary diversity than those over the age of 30 years. This might be because older women are more likely to practice food taboos, which may be due to their strong adherence to their descendants’ social cultures [20]. Pregnant women with a low monthly average family income (under 3000 birr) were less likely to practice good dietary diversity than those with a higher average monthly family income (above 4500 birr). This finding is similar to previous studies’ findings [14, 15]. This suggests that the Afar people, who faced food scarcity, were vulnerable to poor dietary diversity. Their pastoralist lifestyle, which is primarily based on livestock, may be contributing to their poor dietary diversity [19]. Pregnant women who had one antenatal care visit were also less likely to consume the minimum dietary diversity than those who had four or more. This could be because more antenatal care visits are linked to increased awareness of the benefits of dietary diversity. This evidence conforms to earlier studies [14, 15]. In this study, the minimum dietary diversity did not have a significant association with educational level, occupation, and eating frequency per day. This evidence is contradicted with previous studies [14–16].

This study is a cross-sectional study, so it is difficult to infer a causal association. However, adding an exploratory study to a quantitative study aids in the discovery of additional information unavailable from the quantitative study. The open-ended questionnaire allowed participants to report any foods or beverages they had consumed or unconsumed without restriction. Thus, these questionnaires allowed women to describe their experiences in their own words, but they were vulnerable to social desirability. Using a single 24-h recall period did not reveal an individual's habitual diet, and the amount of food consumed is not indicated by the dietary diversity score. The attainment of pregnant women at antenatal clinics was low, particularly in rural areas, resulting in a smaller number of participants being recruited from these areas. The fact that the sample was limited to a single season may restrict the generalization of the results to subsequent seasons. However, the study provided a new perspective on factors that influence dietary diversity among pregnant women, particularly in the pastoral community of Afar, which is novel and interesting. Therefore, the findings can give useful information for nutrition-sensitive intervention.

## Conclusion

Most study participants consumed less than the minimum dietary diversity. Younger pregnant women had better dietary diversity than older pregnant women. Having a high average family income was associated with good dietary diversity. Those pregnant women, with one antenatal care visit, had less minimum dietary diversity than those who had four or more antenatal care visits. Protein-rich foods (meat and eggs), semisolid foods (porridge and cereal soup), and milk were the most commonly avoided foods by pregnant women. The most common reasons for avoiding these meals were delivery difficulties and fear that they might stick to the fetus's body. To reduce low diet diversity practices, which reflect nutrient deficiencies in pregnant women's diets, public health awareness campaigns at all primary healthcare institutions and community levels should be provided. Antenatal care utilization and reform the economy are also needed. Furthermore, pregnant women who do not consume protein-rich foods (meat and eggs) and semi-solid foods (porridge and cereal soup) should receive comprehensive health education, especially older pregnant women. This advice is applicable only in Ethiopia because the data in this study only apply to similar populations, not to places outside Ethiopia or major cities.

## Data Availability

The datasets used for analysis during the current study are available from the corresponding author on reasonable request.

## Abbreviations

FGD: Focus group discussion
SPSS: Social science statistical package
COR: Crude odd ration
AOR: Adjusted odd ratio
CI: Confidence interval
SD: Standard deviation
